# Insertional Oncogenesis by Non-Acute Retroviruses: Implications for Gene Therapy

**DOI:** 10.3390/v3040398

**Published:** 2011-04-15

**Authors:** Hung Fan, Chassidy Johnson

**Affiliations:** Department of Molecular Biology and Biochemistry, Cancer Research Institute, University of California, Irvine, CA 92697, USA; E-Mail: chassidj@uci.edu

**Keywords:** retrovirus, proto-oncogene, oncogenesis, retroviral vector, oncogene, non-acute retrovirus, gene therapy

## Abstract

Retroviruses cause cancers in a variety of animals and humans. Research on retroviruses has provided important insights into mechanisms of oncogenesis in humans, including the discovery of viral oncogenes and cellular proto-oncogenes. The subject of this review is the mechanisms by which retroviruses that do not carry oncogenes (non-acute retroviruses) cause cancers. The common theme is that these tumors result from insertional activation of cellular proto-oncogenes by integration of viral DNA. Early research on insertional activation of proto-oncogenes in virus-induced tumors is reviewed. Research on non-acute retroviruses has led to the discovery of new proto-oncogenes through searches for common insertion sites (CISs) in virus-induced tumors. Cooperation between different proto-oncogenes in development of tumors has been elucidated through the study of retrovirus-induced tumors, and retroviral infection of genetically susceptible mice (retroviral tagging) has been used to identify cellular proto-oncogenes active in specific oncogenic pathways. The pace of proto-oncogene discovery has been accelerated by technical advances including PCR cloning of viral integration sites, the availability of the mouse genome sequence, and high throughput DNA sequencing. Insertional activation has proven to be a significant risk in gene therapy trials to correct genetic defects with retroviral vectors. Studies on non-acute retroviral oncogenesis provide insight into the potential risks, and the mechanisms of oncogenesis.

## 1. Introduction

Retroviruses have historically been of interest because they induce cancers in animals; some of the fundamental principles of molecular cancer biology were first discovered through studies on these viruses (e.g., the discovery of oncogenes and proto-oncogenes). Retroviruses of humans are associated with human T-cell leukemia (HTLV-I) and AIDS (HIV-1). Very recently new human retroviruses have been discovered (e.g., XMRV) [1,2], and their relationships to human disease are under debate and active investigation [1,3–10]. This review will focus on non-acute retroviruses—those that induce tumors indirectly through activation of cellular genes.

## 2. Retrovirus Structure and Replication

**Figure 1.**
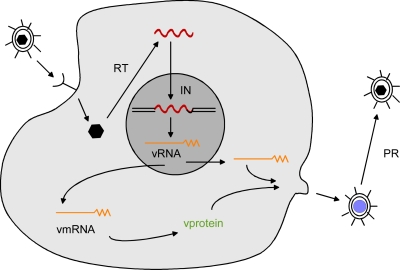
The retrovirus life cycle. See text for details.

Retroviruses have been studied intensively for the past forty years, and many details of their structure and replication have been elucidated (reviewed in Coffin *et al.* [11]). A brief summary is provided here. Retroviruses are enveloped RNA viruses that carry two identical copies of genomic RNA in the virion. They have relatively simple genomes (8–12 Kb in length); the genomic RNA is positive stranded and resembles cellular mRNA in that is capped at the 5′ end and polyadenylated at the 3′ end. All retroviruses contain at least three genes: *gag* that encodes the viral core proteins (matrix [MA], capsid [CA] and nucleocapsid [NC]), *pol* that encodes the viral enzymes (protease [PR], reverse transcriptase [RT] and integrase [IN]), and *env* that encodes the proteins of the viral envelope (surface [SU] and transmembrane [TM]). The retrovirus life cycle is illustrated in Figure 1. When retroviruses infect cells, they bind to cell surface receptors via the envelope SU protein. The bound virus then enters the cell either by receptor-mediated endocytosis or fusion at the plasma membrane. The result is viral cores in the cytoplasm. Reverse transcriptase is activated within the cores, where it uses the viral RNA as a template for synthesis of linear double-stranded viral DNA. The viral DNA is then transported to the nucleus, where it is integrated into the host chromosome by the viral integrase, to form the provirus. Integration of viral DNA into the host DNA occurs at multiple (almost random) sites, although for various retroviruses there is some preference for integration. For instance, murine leukemia viruses tend to favor viral DNA integration near transcriptional start sites [12]. The provirus is transcribed by cellular RNA polymerase II, yielding a viral transcript that is identical to genomic RNA. The viral transcript is exported to the cytoplasm with or without mRNA splicing. In the cytoplasm, spliced viral mRNA is translated into a polyprotein precursor for envelope protein. Some of the cytoplasmic unspliced viral RNA is translated into precursor polyproteins for Gag or Pol (a Gag or Gag-Pol polypeptide). The viral polyproteins combine with viral RNA to form virus particles that bud from the cell surface. The initially released viral particles are immature and non-infectious. Maturation results from viral protease cleavage of the viral polyproteins in released virions. Retroviral infection is typically not lytic—the end result of infection is a cell stably producing virus particles.

**Figure 2.**
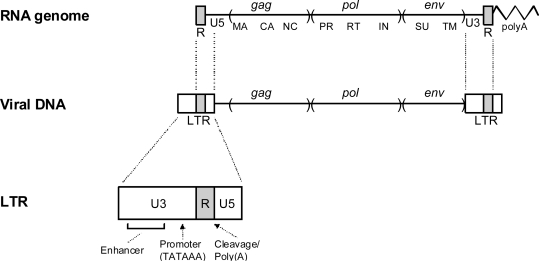
Retroviral LTRs. The relationship of retroviral RNA to the reverse transcribed DNA is shown. The viral RNA contains short direct repeats at either end (R), and the viral DNA contains long terminal repeats. Subdivision of the LTR into U3, R and U5 regions is shown at the bottom.

The process of reverse transcription results in a viral DNA that is somewhat longer than the template viral RNA, due to the presence of long terminal repeats (LTRs) at either end of the viral DNA (Figure 2). The LTRs are subdivided into three regions, according to the region of the viral genome from where they are encoded. U3 sequences are encoded in viral RNA sequences Uniquely at the 3′ end of the genome, U5 sequences are encoded from sequences Uniquely at the 5′ end of the genome, and R sequences are encoded by RNA sequences that are Repeated at either end of the genome. The LTRs carry important transcriptional signals. These include a cleavage/polyadenylation site in the R region and a basal promoter and enhancers in U3. Transcription is initiated at the U3-R boundary in the upstream LTR, and cleavage/polyadenylation takes place at the R-U5 boundary in the downstream LTR.

## 3. Retroviral Oncogenesis: Acute Transforming *vs.* Non-Acute Retroviruses

Retroviruses cause a variety of tumors in animals and humans, ranging from solid tumors such as carcinomas and sarcomas through hematopoietic neoplasms such as leukemias and lymphomas. They can be divided into two classes: *acute transforming retroviruses* and *non-acute* retroviruses [13]. As the name implies, acute transforming retroviruses induce tumors rapidly, while non-acute retroviruses induce tumors with a longer latency. Acute transforming retroviruses were among the first retroviruses to be discovered—Rous sarcoma virus (RSV), a virus that causes fibrosarcomas in chickens, is the prototypical virus of this class. A common feature of acute transforming retroviruses is that they carry additional genetic information—viral oncogenes [13,14]. The viral oncogenes endow acute transforming retroviruses with the ability to induce tumors rapidly, and acute transforming retroviruses frequently can alter the growth properties of infected cells in tissue culture (cell transformation) [15]. The oncogene of RSV is called *v-src*; it encodes a tyrosine-specific protein kinase [16–19]. Other acute transforming retroviruses carry different oncogenes; for instance avian MC29 virus that induces acute myeloid tumors in chickens carries an oncogene called *v-myc* [20] and avian erythroblastosis virus carries two oncogenes, *v-erbA* and *v-erbB* [21]. Approximately 25 viral oncogenes have been discovered; in some cases more than one acute transforming retrovirus carries the same or a similar oncogene.

A seminal finding was that retroviral oncogenes are actually derived from normal cell genes [22]. The normal cell counterparts of retroviral oncogenes are called cellular proto-oncogenes. For instance *v-src* was derived from the proto-oncogene *c-src*. As a group the cellular proto-oncogenes encode proteins that function in positive (but regulated) stimulation of cell growth or division and can promote cell survival. Viral oncogene proteins differ from the corresponding cellular proto-oncogene proteins in various ways, but a common theme is that the viral oncogene proteins cause unregulated stimulation of cell growth/division compared to their cellular proto-oncogene protein counterparts. Many cellular proteins in key signal transduction pathways were first discovered as cellular proto-oncogene counterparts of viral oncogenes. These include the cellular Ras [23–25], Raf [26,27], Myc [28,29], Fos [30,31], Jun [32,33], and Akt [34,35] proteins to name a few. The discovery of cellular proto-oncogenes was of fundamental importance to cancer research, since it became apparent that non-viral cancers frequently have activating mutations of proto-oncogenes [36,37] or they over-express them through gene amplification [38–40] or chromosomal translocation [41–43]. These were the first kinds of genetic mutations in human cancers to be identified.

Non-acute retroviruses do not carry viral oncogenes, and they do not transform cells in culture. They induce tumors more slowly than acute transforming retroviruses. Non-acute retroviruses have the standard genome organization of retroviruses, while most acute-transforming retroviruses are replication-defective because they have substituted oncogene sequences in place of viral genes. These viruses must be co-infected with a related “helper virus” that provides the viral structural proteins to make infectious particles. Examples of non-acute retroviruses include avian leukosis viruses that induce lymphoid tumors in chickens, murine leukemia viruses (MuLVs) that induce leukemias, murine mammary tumor virus (MMTV) that induces mammary carcinomas, and feline leukemia viruses (FeLVs) [13]. The mechanisms by which these non-acute retroviruses induce tumors will be subject of this review.

## 4. Insertional Activation of Proto-Oncogenes

**Figure 3.**
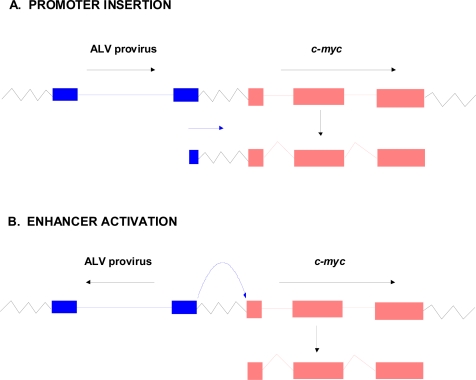
Insertion activation of *c-myc* in avian leukosis virus (ALV)-induced B-lymphomas. (**A**) Promoter insertion activation of *c-myc* as reported by Hayward *et al.* [42]. (**B**) Enhancer activation of *c-myc* (one of the configurations) reported by Payne *et al.* [43].

The landmark study that elucidated the basic principle of how non-acute retroviruses induce tumors was published by Hayward *et al.* in 1981 [44]. These investigators studied B-lymphomas in chickens induced by avian leukosis virus (ALV). They carried out Southern blot and Northern blot analyses of multiple ALV-induced tumors using two hybridization probes for ALV: a probe representative of the entire ALV genome (cDNA_rep_) and another one that contained sequences only from the 5′ end (the R and U5 regions of the LTR-cDNA_5′_). Analysis of Southern blots of tumor DNAs with the ALV cDNA_rep_ probe indicated that while viral DNA could be detected in all tumors, there were frequently missing viral sequences. Likewise when tumor RNAs were studied by Northern blotting with cDNA_rep_, not all tumors showed evidence for viral RNA transcripts. On the other hand, all of the tumors showed transcripts that could be detected by ALV cDNA_5′_. The fact that the transcripts could be detected by cDNA_5′_ but not cDNA_rep_ led Hayward *et al.* to propose that they represented transcripts that initiated in the downstream LTR and read into adjacent host sequences [44]. Moreover, the fact that the transcripts detectable by cDNA_5′_ from the different tumors were of approximately the same size led the investigators to propose that viral DNA was being inserted into the same chromosomal locations in the different tumors—the length of the transcripts would be determined by the site of proviral insertion and the relative position of the cleavage/polyadenylation signals in the host cell DNA. They proposed that the inserted proviral DNA was leading to over-expression of the host cellular gene by read-through transcription from the viral LTR, a process termed “promoter insertion” (Figure 3A). Over-expression of the cellular gene was ultimately responsible for the leukemogenesis.

Proof of the promoter insertion model for ALV leukemogenesis was obtained when the activated cellular gene was identified. Hayward *et al.* tested the hypothesis that the activated cellular gene might be a proto-oncogene, *i.e.*, the cellular homolog of a known viral oncogene [44]. They were able to demonstrate that these tumors contained ALV DNA inserted next to the *c-myc* proto-oncogene. This was accomplished by using a *v-myc* hybridization probe that would cross-hybridize with *c-myc* sequences. They showed that the RNA transcripts in ALV-induced tumors that could hybridize with cDNA_5′_ would also hybridize with the *v-myc* hybridization probe, and that the *c-myc* genes in the tumors also had insertions of ALV DNA.

The promoter insertion mechanism of ALV leukemogenesis also provided a conceptual framework for understanding the relatively long latency of the disease. Since retroviruses integrate proviral DNA at virtually random sites throughout the genome, the likelihood of an insertion in the vicinity of *c-myc* in any infected cell would be quite low. Multiple rounds of infection (and time) would be necessary before an ALV provirus inserted next to *c-myc* in one infected cell. That infected cell would then receive the enhanced growth signals and develop into the tumor. For this reason, tumors induced by non-acute retroviruses are also typically monoclonal or oligoclonal outgrowths of a single (or small number) of infected cells. However, as discussed below, the long latency of disease is affected by other factors as well.

The studies of Hayward *et al.* [44] were followed up by another group in which ALV-induced B-lymphomas induced in chickens with a different genetic background were examined [45]. They confirmed integration of ALV proviruses adjacent to *c-myc* in tumor DNAs, as well as the over-expression of *c-myc* RNA. However, not all of the tumors had proviral integrations compatible with promoter insertion activation of *c-myc* (Figure 3B). In some cases the ALV provirus was inserted upstream of *c-myc* but in the opposite transcriptional orientation, or downstream from *c-myc* but in the same orientation. These cases ultimately have been ascribed to the strong ALV enhancers in the LTR activating the endogenous *c-myc* promoter, resulting in over-expression of a normal *c-myc* transcript. T-lymphomas induced in mice by Moloney murine leukemia virus (M-MuLV) have also been found to result from activation of *c-myc* [46,47], and in this case the predominant mechanism is enhancer activation [46]. In addition, other M-MuLV-induced T-lymphomas result from activation of novel proto-oncogenes (see following section).

Another group studied tumors induced by ALV, but in a line of chickens resistant to lymphoma, where erythroleukemias developed instead [48]. In this case, promoter insertion of a different proto-oncogene *c-erbB* (a.k.a. epidermal growth factor receptor) occurred. In these tumors proviral insertion was into the coding sequences of *c-erbB*, and transcription initiated in the upstream ALV LTR, with read-through from the downstream LTR into *c-erbB* [49,50]. This resulted in a fusion transcript that was further processed by mRNA splicing to encode a novel protein containing viral Gag and Env sequences fused to a truncated EGF receptor.

Overall, the general mechanism for oncogenesis by non-acute retroviruses can be considered to be LTR-activation of proto-oncogenes. This includes classical promoter insertion as well as enhancer activation. Depending on the retrovirus and the biological system, one or the other, or both mechanisms may predominate.

## 5. Discovery of New Proto-Oncogenes by Studying Insertional Activation

Other investigators studied tumors induced by other non-acute retroviruses using the same conceptual framework as developed by Hayward *et al.* However, in some cases many if not all of the tumors did not show proviral insertions in the vicinity of proto-oncogenes known at the time. Nusse *et al.* studied mammary tumors induced by MMTV in mice [51]. They identified tumors with relatively low numbers of inserted proviruses by Southern blotting, and then they cloned all of the proviruses from such a tumor. The adjacent cellular sequences from the different proviruses were then used as hybridization probes in Southern blots of other MMTV-induced tumors, in search of common insertion sites (CISs). It was hypothesized that a CIS would be in the vicinity of a cellular gene that was activated by proviral insertion. In any given tumor, all of the integrated proviruses would not necessarily represent a CIS; indeed only one or a small number would likely be. Hence the searches for CISs began with tumors containing small numbers of inserted proviruses, to increase the probability that any cloned provirus represented a CIS. In the case of MMTV-induced mammary tumors, the CISs were designated *int-1* and *int-2* [51–53], and subsequent studies of MMTV led to identification of *int-3* [54]. *Int-1* is a founding member (*Wnt1*) of the *Wnt* family of growth factor receptors, which are components of the *Wnt-beta-catenin* signaling pathway [55]. This pathway is frequently dysregulated in many human epithelial tumors such as colon cancer [56]. *Int-2* has been subsequently found to be the same as fibroblast growth factor 3 (Fgf3), and *int-3* has been found to be *Notch4* [55].

Similar studies on various MuLV strains in mice and rats have led to identification of other novel proto-oncogenes through identification of CISs (Table 1). These include *pim-1* [57], *pim-2* [58], *Mis-1* [59], *Spi-1* [60,61] and *Fli-1* [62]. Pim-1 is a serine-threonine kinase, and Spi-1 (PU.1) and Fli-1 are transcription factors of the Ets family. In each of these cases, this was the first identification of these genes, which have subsequently been found to play important roles in normal physiology, and to be dysregulated in certain cancers. In addition to the CISs identified from tumors induced by exogenous retroviruses, Copeland, Jenkins and co-workers have employed recombinant inbred mouse strains that have high frequencies of leukemia due to spontaneous activation of endogenous MuLVs—this has led to identification of another set of CISs termed endogenous virus insertions (*evi*-1, 2, *etc.*) [63].

In general, a given non-acute retrovirus will induce a specific type of tumor, or a restricted range of tumor types (e.g., T-lymphomas for M-MuLV, myeloid and erythroid leukemias for Friend MuLV). Correspondingly, the tumors induced by a particular virus will typically show activation of a limited number of proto-oncogenes. For instance, T-lymphomas induced by M-MuLV predominantly show activation of *c-myc*, *pim-1* and/or *pim-2* [46,58]. In contrast, erythroid leukemias induced by Friend MuLV predominantly show activations of *fli-1* [62], and myeloid tumors in recombinant inbred mice show activations of *evi-1* [64]. This may reflect the relative abilities of particular proto-oncogenes to contribute to malignancy in different cell types when they are over-expressed. The genetic background of the host also affects the pattern of insertional activation (discussed in Section 7).

## 6. LTRs as Determinants of Disease Specificity

Related retroviruses can differ in their abilities to induced tumors, as well as the kinds of tumors induced. Generation of molecular chimeras between related viruses identified the LTRs as primary pathogenic determinants. For instance, the LTR from an oncogenic MuLV (e.g., Gross MuLV) could confer leukemogenicity to a related weakly leukemogenic Akv-MuLV [67,68]. Likewise the LTR from Friend MuLV that induces erythroleukemia could convert M-MuLV from inducing T-lymphoma to erythroleukemia [69]. This was extended to show that just the enhancer elements in the U3 region of the LTRs were sufficient to switch the disease specificity [70]. The disease specificity of the Friend *vs.* Moloney LTRs was also correlated with the relative transcriptional activities of these LTRs in lymphoid *vs.* erythroid/myeloid cells [71]. Enhancer sequences consist of binding motifs for sequence-specific transcription factors. For some retroviruses, these are tandemly repeated which makes binding stronger.

The importance of the LTRs in oncogenicity of non-acute retroviruses can be understood in the context of LTR-activation of proto-oncogenes. In order for a non-acute retrovirus to induce oncogenic transformation of a particular differentiated cell, it must have an LTR that can transcriptionally activate proto-oncogenes in that cell (either by promoter insertion or enhancer activation). The enhancer sequences of the LTR bind cellular transcription factors, and they are frequently cell-specific, with binding motifs for factors that may be highly expressed in particular differentiated cells. Thus the ability of a retrovirus to activate proto-oncogenes and induce tumors will depend on the relative strength of the enhancer sequences, and the cells in which they are active.

## 7. Cooperation among Activated Proto-Oncogenes

In some cases tumors induced by non-acute retroviruses have shown evidence for activation of more than one proto-oncogene in the same tumor—e.g., *int-1 (Wnt-1)* and *int-2* in MMTV-induced mammary tumors [72] and *pim-1* and *c-myc* in M-MuLV-induced tumors [46]. This could reflect cooperation of the two proto-oncogenes (either within the same tumor cell or between adjacent cells), or simply two independent tumors in the same mass. This has been addressed by transplanting the original tumors into recipient animals and analyzing the secondary tumors for the presence (or not) of both proto-oncogene activations [72,73]. The results indicated that the tumors induced by these viruses are generally oligonal collections of independent tumors, some of which contain activation of one proto-oncogene while others contain activation of the other. On the other hand, in the case of MMTV-induced tumors with *int-1* and *int-2* insertions, transplantation into recipient mice resulted in maintenance of independent oligoclonal populations with *int-1* and *int-2* insertions when the tumors were hormone-dependent [72]. This suggests that in MMTV-induced hormone-dependent tumors, cooperation between tumor cell sub-populations with activated *int-1* and *int-2* genes may be occurring.

Intracellular cooperation among proto-oncogenes in MuLV leukemogenesis was demonstrated by Berns and co-workers in transgenic mice over-expressing *pim-1* under control of the immunoglobulin heavy chain enhancer (Eμ) [74]. The transgenic mice developed T-cell lymphomas slowly (7 months) and at a low frequency (5–10%). However, when these mice were infected with M-MuLV all of them developed tumors and much more rapidly (7–8 weeks). The resulting tumors were found to harbor M-MuLV insertions near *c-myc* or the related *n-myc* proto-oncogene [74]. This provided strong evidence for cooperation between *pim-1* and *myc* family proto-oncogenes in development of T-lymphoma. Cooperation between *pim-1* and *myc* proto-oncogenes was again observed when mice transgenic for *c-myc* driven by the Eμ promoter/enhancer (Eμ-*myc*) were infected with M-MuLV [75]. Acceleration of leukemogenesis (T- or B-lymphoma) occurred, and a substantial fraction of the tumors showed proviral insertions next to *pim-1.* Other tumors showed novel CISs [75], leading to the identification of new proto-oncogenes (e.g., *bmi-1*). This discovery process is now referred to as retroviral tagging.

Retroviral tagging in double and triple transgenic mice has been used to identify proto-oncogenes active in particular pathways. For instance Eμ-*myc/Pim-1^−/−^* mice were infected with M-MuLV with the goal of identifying proto-oncogenes that could substitute for *Pim-1* [76]; the predominant activated proto-oncogene was *Pim-2,* indicating that it cooperates with *c-myc* in the absence of *Pim-1*. This rationale was extended to studies in Eμ*myc/Pim-1^−/−^/Pim-2^−/−^* mice where *Pim-3* was identified [77]. Retroviral tagging studies in transgenic mice over-expressing *myc* from other T-cell specific promoters have identified other cooperating proto-oncogenes, including *Notch1* [78] and *Runx2* [79,80]. More recently the same strategy has been used by others to identify genes that can collaborate with *myc* and *Runx2* [81] or complement deficiencies in multiple cycling-dependent kinase inhibitors [82].

Recently retroviral tagging has been extended to identifying potential cooperating proto-oncogenes in solid tumors [83]. Inactivations of the APC tumor suppressor protein are important for colon cancer development [56]. Mouse colon epithelial cells from mice containing an APC mutation (Min) were infected *in vitro* with the MSCV strain of MuLV. Infected cells from Min mice formed colonies in soft agar while those from wild-type APC did not. Colony formation in agar is one property associated with malignant transformation. CISs in the agar colonies identified several proteins that can apparently collaborate with mutated APC in transformation [83].

## 8. Insertional Activation in Multi-Step Carcinogenesis and Tumor Progression

It is now well-understood that cancer is a multi-step process, with multiple genetic and biochemical changes taking place within a developing tumor cell. While insertional activation of proto-oncogenes by non-acute retroviruses has traditionally been viewed as an initiating or early event in tumor development, it can also participate in later steps in tumorigenesis. One interesting case was studies on B-lymphomas induced by Abelson MuLV. Ab-MuLV is an acute transforming retrovirus whose replication-defective genome carries the *v-abl* oncogene. In order to efficiently infect mice and induce lymphomas Ab-MuLV must be co-infected with a helper MuLV—originally M-MuLV. However when a different helper MuLV was employed (from an endogenous MuLV), the rapid leukemogenicity of Ab-MuLV was lost [84]. Chimeras between the two helper MuLVs indicated that the M-MuLV LTR was necessary for Ab-MuLV leukemogenesis. This led Poirier *et al.* to test the hypothesis that the M-MuLV helper was activating proto-oncogenes that cooperated with the v-Abl protein to produce the tumors [85], and they indeed indentified a CIS (*ahi-1*) in some of the tumors. This suggested that the M-MuLV helper is cooperating with v-Abl oncogene protein in inducing the rapid B-lymphomas. M-MuLV insertions into *ahi-1* typically are downstream of the last exon, but in some cases they generate C-terminal truncations of the putative protein [86].

The ability of activated proto-oncogenes to participate in later steps in M-MuLV leukemogenesis has been studied by Tsichlis and co-workers. When M-MuLV-induced lymphomas from rats are cultured *in vitro*, they tend to acquire additional copies of M-MuLV proviruses. Bear *et al.* [87] hypothesized that these could represent activation of “tumor progression loci”, and they discovered several CISs in the cultured cells (*tpl-1, tpl-2*) [87,88]. They found evidence for insertional activation of these loci in tumors as well. These same workers also investigated the possibility that MCF recombinant viruses that arise in MuLV-infected mice could also be contributing to leukemogenesis by insertional activation. *In vitro* infection of M-MuLV-induced tumor cells with MCF recombinants and culture in the absence of growth factors led selection of cells with viral integrations into new CISs (*gfi-1, gfi-2*) [89,90]. *Gfi*-1 encodes a novel DNA binding protein, while *gfi*-2 encodes the IL-9 receptor. Thus activation of proto-oncogenes can participate in later stages of oncogenesis, downstream of other proto-oncogene activations, viral oncogenes, or potentially non-viral oncogenic events.

While MuLVs do not morphologically transform cells in culture, Heard *et al.* [91] have observed multi-stage changes culminating in development of myeloid leukemia cells when long term bone marrow cell cultures were infected *in vitro* with F-MuLV. With continued passage, cells in the culture progressed through stages of (1) enhanced responsiveness to macrophage colony stimulating factor (M-CSF/CSF-1), (2) growth factor independence, and ultimately (3) acquisition of tumorigenicty. Tumors induced in this system showed two novel CISs, *Fim-1 and Fim-2* [92]; *Fim-2* was later found to be the structural gene for M-CSF [93].

## 9. Other Mechanisms of Insertional Oncogenesis

The most common mechanism of oncogenesis by non-acute retroviruses is transcriptional activation of proto-oncogenes. However proviral insertions can have other effects that result in oncogenic stimuli. One example is in erythroleukemias induced by the Friend virus complex—an acute transforming retrovirus (SFFV) as well as a helper virus (F-MuLV). Erythroleukemia cell lines established from these tumors show inactivation of the p53 tumor suppressor gene due to insertion of an SFFV provirus [94,95]. In most of the cell lines, the normal p53 gene was also lost, resulting in lack of functional p53. Insertional inactivation of the NF-1 tumor suppressor gene has also been found in myeloid leukemias arising in BXH-2 recombinant inbred mice [96]—the CIS originally termed *evi-2* was found to be in the NF-1 gene.

Another example is myeloid tumors induced by M-MuLV in adult Balb/c mice primed with pristane. Pristane treatment results in inflammation and myeloid cell expansion. The resulting tumors show insertion of M-MuLV proviruses within the 3rd or 4th introns of the *c-myb* proto-oncogene in the same transcriptional orientation [97,98]. This leads to expression of a hybrid transcript initiated in the M-MuLV provirus with readthrough into the downstream *c-myb* sequences; mRNA splicing leads to a truncated *c-myb* mRNA and a *c-myb* protein lacking an N-terminal negative regulatory domain [97]. This results in a constitutively active c-myb protein (transcription factor), which contributes to development of the myeloid tumors. Thus proviral insertion can lead to alteration (truncation) of a proto-oncogene protein that results in oncogenic activation. Another interesting feature of this system is that administration of pristane, which elicits a strong macrophage response, is necessary for development of the myeloid tumors [99]. The pristane is inducing inflammation and mitogenesis in myeloid cells that presumably cooperates with the oncogenic effects of the truncated c-Myb protein. Indeed, the same M-MuLV insertions into *c-myb* can be detected in lymphoid tissue from other strains of mice inoculated as newborns in the absence of pristane, but the T-lymphomas that develop do not show *c-myb* insertions [100]. Thus the truncated c-Myb requires additional mitogenic stimuli to manifest its oncogenic effect, and this protein may not be effective in lymphoid cells. In fact in erythroleukemias induced by ALV in chickens, the activated form of the EGF receptor (*c-erbB*) is truncated at the amino terminus (extracellular growth factor binding domain), similar to the activated v-erbB oncoprotein of avian erythroblastosis virus [49].

Another very interesting mechanism of insertional activation is in chicks infected embryonically and then post-hatching with ALV. Some of these animals developed B-lymphomas that frequently contained proviral insertions at *c-myc* as well as a novel CIS designated *bic-1* [101]. The ALV insertion enhanced expression of the *bic-1* transcript, but at the time, the mechanism of action of *bic-1* was unclear, since it could not encode any protein. More recently, *bic-1* has been found to be the precursor of miR-155, a microRNA (miRNA) whose over-expression has been observed in human cancers as well [102]. Over-expression of miR-155 leads to down-regulation of target transcripts— tumor suppressors such as JARID2/jumonji [103]. Indeed, *bic-1* is the first oncogenic miRNA to be discovered. Similarly a CIS previously identified in RadLV—induced T-lymphomas in mice (Kis2) has been found to encode the precursor for the miRNA cluster, miR-106–363 [104]. Over-expression of miR-106–363 could induce anchorage-independent growth, and human T-lymphomas also showed over-expression of this miRNA cluster [104]. In SL3-3 MuLV-induced T-lymphomas, proviral insertion and over-expression of the miR-106a cistron has also been reported [105].

Another recent study has characterized T-lymphomas in mice induced by the SL3-3 strain of MuLV [106]. A frequent CIS in these tumors was found to be the *Gfi-1* proto-oncogene (see above). Interestingly, in some cases, the proviral insertion was into the 3′ untranslated region of the *gfi-1* RNA, resulting in a truncated transcript. Tumors with such transcripts showed a high level of Gfi-1 protein, while tumors that did not have proviruses inserted in the 3′ UTR did not. Dabrowska *et al.* provided evidence that the 3′ UTR of *Gfi-1* contains target sites for several miRNAs (including miR-155), and that the proviral insertions would uncouple the Gfi-1 coding sequences from the miRNA binding sites [106]. Thus over-expression of Gfi-1 protein could result from truncation of miRNA binding sites.

## 10. Insertional Mutagenesis in the Age of Genomics

Original methods to identify CISs were laborious and time consuming, involving Southern blotting, screening DNA libraries, genome walking and extensive cloning. Due to the large distances over which a provirus can act on a gene and the limited knowledge of the mouse genome, identification of the proto-oncogenes in the vicinity of a CIS was challenging. Identification of CISs and retroviral tagging drastically changed with the development of PCR-based strategies (inverse and splinkerette-based PCR) for cloning host-virus junctions, along with sequencing of the mouse genome. The host DNA on cloned host-virus junction fragments could be aligned with the mouse genome, yielding precise location of the insertions sites. Thus high-throughput studies could be performed with hundreds of insertion sites identified in a single study. Copeland and co-workers were the first to use this approach on leukemias arising in recombinant inbred mice (AKXD and BXH-2), identifying more than 90 potential CISs in one experiment [107]. Other investigators have carried out similar studies, using retroviral tagging in wild-type or in tumor-prone transgenic/knock-out mice. This allowed identification of novel CISs involved in development of particular tumor types, or that collaborate with known oncogenic pathways [77,81,108–110]. Currently, over 600 CISs have been identified. For a compilation of these sites see the Mouse Retrovirus Tagged Cancer Gene Database [65,66].

Very recently the advent of high throughput DNA sequencing has further increased the rate at which CISs can be identified. Host-viral junction fragments are PCR-amplified from tumor cells and then directly subjected to deep sequencing with the goal of identifying as many host cell insertion sites as possible. In a recent study, more than 9000 insertions from 476 lymphomas were identified [82]. Data from retroviral tagging screens are now being compared with other cancer genome screens (e.g., comparative genomic hybridization) [111], providing a rich database for genetic changes in different cancers.

Examination of CISs in MuLV-induced tumors has also suggested involvement of additional miRNAs in tumorigenesis. An informatic search of the RTCGD database identified 19 CISs that were within 10 Kb of an miRNA [112]. Some of these insertions could affect expression of an miRNA, similar to the activation of *bic-1*/miR155 in ALV-induced lymphomas.

## 11. Insertional Mutagenesis and Gene Therapy

**Figure 4.**
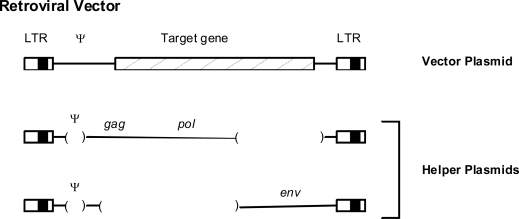
Retroviral vectors. Organization of a retroviral vector as well as two helper plasmids to produce the viral proteins are shown. Ψ, the packaging sequences on viral RNA. In some cases the helper plasmids are stably expressed in a packaging cell, or in other cases they are co-transfected with the vector plasmid.

Retrovirus-based vectors have been employed in human gene transfer experiments to introduce therapeutic molecules into cells to combat various diseases. The generation and use of retroviral vectors has been reviewed extensively [113]. Briefly, retroviral vectors are generated by recombinant DNA manipulations on plasmids containing a retroviral provirus. Internal viral coding sequences are substituted with DNA encoding a gene of interest; the viral LTRs and encapsidation (psi) signals for incorporation of viral RNA into particles are retained (Figure 4). Retroviral particles are obtained by introducing the vector DNA into “packaging” cells that express viral structural proteins from mRNAs that cannot themselves be packaged, or transiently co-transfecting cells with vector and helper plasmids. The transfected packaging cells will then produce viral particles that contain the vector sequences as RNA. These vector particles can then be used to infect target cells where reverse transcription and integration of the vector genome takes place, followed by expression of the vector. Retroviral vectors have the advantages that they integrate their DNA into the genomes of the infected host cells (transduction). Thus the genetic information for therapeutic molecules will permanently integrate into the target cells, which can lead to stable and prolonged expression. Retroviral vectors based on gammaretroviruses such as murine leukemia viruses were some of the first vectors employed in human gene transfer experiments, and they are still being used. More recently vectors based on lentiviruses such as HIV-1 have been employed [114].

The first human gene transfer trial used an MuLV-based vector expressing adenosine deaminase (ADA) for treatment of individuals with hereditary severe combined immuno-deficiency (SCID-ADA) that resulted from deficiency of ADA. The initial experiment involved *ex vivo* transduction of T-lymphocytes from the patients, followed by infusion of the transduced cells [115]. Transduced T-cells could be persistently detected in the patients receiving them, and there was evidence for clinical benefit—improvement of immunological status. Despite the theoretical concern that the transduced vectors might cause malignancies by insertional activation of proto-oncogenes, the two patients in the first trial did not show any evidence of malignancies. Subsequent trials to treat SCID-ADA have involved transduction of hematopoietic precursors (CD34+) *ex vivo* with ADA-expressing retroviral vectors; in total more than 30 patients have been treated worldwide, with correction of the immunodeficiency in many, and no malignancies [116].

A watershed event for the gene therapy field occurred in clinical trials involving X-linked SCID—a genetic deficiency of the common gamma chain for growth factor receptors [117,118]. Hematopoietic progenitor cells (CD34+) from X-SCID patients (n = 20, results combined from two clinical trials in France and the UK) were transduced *ex vivo* with an MuLV-based vector expressing the common gamma chain and then infused into the patients. The transduced cells established and corrected the immunologic deficiency in 19 of 20 patients. However, five of the treated patients ultimately developed T-cell leukemia [119,120]. Analysis of the leukemic cells indicated that they shared a CIS at the cellular proto-oncogene LMO2 [121]. Additional integrations were observed at NOTCH1, CDKN2A, STIL-TAL1, CCND2 and BMI1 [120,122]. LMO2 had previously been found to be activated by chromosomal translocation in human T-cell leukemia [123]. In the X-SCID patients the MuLV LTR of the gene transfer vector was activating LMO2 expression. Thus the theoretical concern of insertional oncogenesis in gene transfer trails with retroviral vectors was confirmed. Recently a patient in an analogous trial to correct the genetic defect in Wiscott-Aldrich syndrome also developed a T-cell leukemia in which insertional activation of LMO2 was observed [124,125]. Likewise, a gene therapy trial using an MuLV-based vector to correct the genetic defect of X-linked chronic granulomatous disease resulted in myelodisplasia (a preleukemic syndrome) associated with insertional activation of the *evi-1* proto-oncogene [126].

Modifications of retroviral vectors to improve their safety have been developed, even before the complications in the X-SCID trial. One common modification is to remove the enhancer sequences from the U3 region of the LTR. This can be accomplished by deleting them from the U3 region of the downstream (but not upstream) LTR, of a plasmid containing a retroviral vector DNA. The U3 sequences in the vector RNA will be derived from the downstream LTR, so after reverse transcription in an infected cell both LTRs will contain the deleted U3 regions and be inactive. Such vectors are referred to as self-inactivating (SIN) vectors [127]. Expression from SIN vectors is obtained by incorporating an internal promoter/enhancer. SIN vectors have the theoretical advantage that the deleted LTRs will not be able to insertionally activate proto-oncogenes. Additional modifications to retroviral vectors have included adding transcriptional insulator sequences to the LTRs or to the internal promoters [128]. Insulators prevent enhancers from activating promoters on the other side of an insulator.

Lentivirus-based vectors are currently of considerable interest since they can infect non-dividing cells. Also lentiviruses (including HIV-1 and animal lentiviruses) do not induce tumors. In addition, while MuLV shows a preference for proviral insertion at or near start sites of transcription [129], HIV-1 shows a preference for inserting into the bodies of genes but not at their promoters [130]. The lack of integration specificity for transcriptional start sites might reduce the likelihood of insertional activation of proto-oncogenes by lentiviral vectors, although enhancer activation of proto-oncogenes can occur over several kilobases of DNA. Lentiviral SIN vectors have also been developed.

Human gene transfer trials with lentiviral vectors are just beginning. One of the first trials has involved use of an HIV-based SIN vector expressing beta-globin to treat patients with beta-thalassemia [131]. In the first patient the vector was successful in correcting the beta-globin deficiency. However clonal dominance of a population of hematopoietic cells occurred in this patient, and the cells over-expressed HMGA2, a protein that is also over-expressed in cancers [132]. This could potentially indicate a pre-neoplastic state or cells with enhanced malignant potential, although there has been no progression to malignancy over 30 months. When the molecular mechanism for the over-expression of HMGA2 was investigated, it was found that the lentiviral vector was inserted downstream of HMGA2, which resulted in readthrough and splicing from HMGA2 mRNA into a cryptic splice acceptor site in a beta-globin insulator of the vector. This resulted in truncation of the HMGA2 mRNA and removal of the binding site for a regulatory miRNA which led to over-expression of HMGA2, analogous to activation of *gfi-1* by SL3-3 MuLV (see Section 9). In fact lentiviral vectors might be more efficient at this mechanism, given their preference for inserting within the coding sequences of genes.

The mechanisms of oncogenesis by non-acute animal retroviruses reviewed here provide useful perspectives for considering safety of retroviral vectors in human gene transfer experiments. Vector-associated malignancies have been observed in correction of three diseases so far, and monoclonal expansion has been observed in the ongoing beta-thalassemia trial. Mechanisms of proto-oncogene activation beyond enhancer/promoter activation need to be considered, and the relative frequencies of different mechanisms may differ for different vectors (gammaretroviral *vs.* lentiviral). The gene therapy field is relatively new, so most of the gene transfer experiments have been monitored for relatively short times. If insertional activation of proto-oncogenes contributes only one preneoplastic event, enhanced development of malignancies might take prolonged periods (decades) to become apparent. Indeed the latency of leukemia development by HTLV-I is decades. It may be impossible to completely eliminate the risk of insertional mutagenesis from retroviral vectors; the relative risks of oncogenesis need to be weighed against the benefits of the gene therapy (e.g., correction of SCID).

## 12. Summary and Perspectives

Studies of retroviruses that lack oncogenes (non-acute retroviruses) have provided important insights into oncogenesis. A common mechanism employed by these viruses is to insertionally activate cellular proto-oncogenes. A frequent process is LTR activation of proto-oncogenes, either by promoter insertion or enhancer activation. Study of different non-acute retroviruses has led to identification of new proto-oncogenes, and this has been accelerated by recent high throughput genomic studies. Study of retroviral oncogenesis in animals has highlighted the fact that oncogenesis is a multi-step process, and that proto-oncogene activation may provide only one or a few steps in this process. Alternate mechanisms of proto-oncogene activation besides LTR activation have also been observed, such as activation by truncation of regulatory domains in either the mRNA or protein. While no evidence for proto-oncogene activation has been observed for the well-characterized human pathogenic retroviruses (HTLV-I and HIV-1), these mechanisms will be important to consider when newly discovered potentially oncogenic human retroviruses are investigated. The mechanisms elucidated by study on non-acute retroviral oncogenesis in animal models also help to frame safety considerations for retroviral vectors in gene therapy trials.

## Figures and Tables

**Table 1.** Common insertion sites (CIS) or activated proto-oncogenes in MuLV-induced tumors^1^.

**VIRUS**	**DISEASE**	**CIS or PROTO-ONCOGENE**
Moloney MuLV	T-lymphoma	
		In mice:
		*c-myc, pim-1, pvt-1/mis-1/mlvi-1, lck, pim-2^a^, n-myc^a^, bmi-1^a^, frat-1^a^, pal-1/gfi-1^a^*
		In rats:
		*c-myc, pvt-1/mis-1/mlvi-1, mlvi-2, mlvi-3, mlvi-4, dsi-1, lck, tpl-1/ets-1^a^, tpl-2^a^, gfi-1/pal-1^a^, gfi-2/IL-9R*
	Myeloid leukemia	*c-myb, mml-1*
AKR MuLV/Gross Virus; SL3-3 MuLV	T-lymphoma	*c-myc, gin-1, n-ras*
RadLV (Radiation leukemia virus)	T-lymphoma	*c-myc, pim-1, vin-1/cyclinD2, notch1, kis-1, kis-2*
Friend MuLV	Erythroleukemia	*fli-1, fre-2*
	Myeloid leukemia	*fis-1, fim-1, evi-1/fim-3, c-fms/fim-2*
Endogenous MuLV (AKXD, BXH-2 recombinant inbred mice)	Myeloid leukemia	*evi-1/fim-3, evi-2, meis-1* and others^1^
	B-lymphoma	*evi-3* and others^1^
Abelson MuLV (contains *v-abl* oncogene)	B-lymphoma	*ahi-1, ahi-2* (M-MuLV helper inserted)
Friend SSFV (SFFV gp52^c^ is an oncogene)	Erythroleukemia	*Spi-1, p53^b^*

1 Data from retroviral tagging of mice genetically predisposed to cancer (e.g., *myc* transgenic mice) are not included here. They can be found in the Mouse Retrovirus Tagged Cancer Gene (RTCG) database [65,66].

a Insertions associated with tumor progression or that collaborate with other proto-oncogene activations;

b Insertion at *p53* inactivates its function;

c gp52 oncogene is a deleted form of endogenous retroviral envelope protein.
